# Psychiatry

**Published:** 1904-09-17

**Authors:** 


### PSYCHIATRY.

**Mental Disease and Leucocytic Changes in the Blood.**—Dr. Lewis C. Bruce, of Perth Asylum, and Dr. A. Peebles have systematically examined the blood of 150 patients admitted to the asylum at Perth during the past three years.[1] Each patient's blood was examined on several (three or more) occasions. With regard to the technique, Cole's system of counting "fields" of the microscope was employed in estimating the frequency of leucocytes, a Thoma-Zeiss hæmocytometer being used. Not fewer than 30 fields were counted for each enumeration, and frequently 60 or more were counted where great accuracy was desired. The staining was done with Louis Jenner's stain. In differential counts the following five varieties of leucocytes were recognised, viz, polymorphonuclear leucocytes, small lymphocytes, large lymphocytes, eosinophile leucocytes, and mast cells. For purposes of comparison, numerous control counts were made from the blood of various members of the staff. In these control cases the leucocytes were 6,000 to 9,000 per c.mm. of blood in young healthy males, and 6,000 to 13,000 in females. Several of the females, however, were anæmic. The average percentages of the different cells were as follows: Polymorphonuclear, 70 per cent.; small lymphocytes, 20 per cent.; large lymphocytes, 8 per cent.; eosinophiles, 1·5 per cent. in the men; and 60 per cent., 30 per cent., 7·5 per cent, and 2 per cent. respectively in the women. Mast cells occasionally occurred, and a percentage of ·5 to 1 per cent. was met with in the control bloods. The following results were obtained: (*a*) In cases of pure acute melancholia leucocytosis was never observed. The leucocytes varied from 7,000 to 13,000 per cmm., the polymor-phonuclear cells from 55 to 70 per cent., the small lymphocytes from 30 to 20 per cent., the large lymphocytes from 5 to 10 per cent. (*b*) The eosinophiles never exceeded 1 per cent., and in a few cases an occasional mast cell was seen. In examining an early case of melancholia which has not fully developed, if hyperleucocytosis is present, and especially if the polymorphonuclear percentage is above 70, the prognosis should be guarded. On the other hand, if the leucocytosis is below 10,000 the prognosis is good for the melancholia. (*c*) Every case of excited melancholia examined showed a high leucocytosis early in the disease, and coinciding with this the percentage of polymorphonuclear cells was frequently above 70. Later in the disease, during relapses (recurrences) a high leucocytosis (20,000 to 30,000) and a high polymorphonuclear percentage (80 per cent.) frequently prevailed. Before the relapse both percentages were low, the rising numbers of leucocytes and polymorphonuclear cells attending the increase of the illness. (*d*) Cases of excitement, which on further examination turned out to be "folie circulaire," often might be mistaken for simple acute mania. All the cases of "folie circulaire" examined showed depression or excitement of the simple melancholia or mania type. During the depressed stage there was a high leucocytosis with a polymorphonuclear percentage of 60 to 70. When apparent recovery followed the leucocytosis still remained high, and when excitement set in the leucocytosis gradually rose, culminating at the height of the mental disturbance (excitement), and then gradually falling. The blood observations showed that "the depression and excitement of cases of 'folie circulaire' are quite different from ordinary attacks of mania and melancholia." (*e*) The leucocytosis of acute continuous mania and recurrent mania in adolescent cases exactly resembles that of excited melancholia, and a notable feature in all three was the persistent high leucocytosis, which remained for months and even years after recovery and discharge from the asylum. (*f*) In cases of recurrent mania (not including cases of alcoholic origin) the leucocytosis rises high during the attack and then falls slightly as the attack passes off, but rarely falls to normal for more than a few days. The polymorphonuclear percentage follows the general curve of the high leucocytosis. Between the excited periods of recurrent mania the large lymphocytes and hyaline cells are much increased, often reaching 20 per cent. (*g*) In acute alcoholic mania (continuous) the changes were identical with those of acute non-alcoholic mania (continuous), suggesting "that alcohol was merely an exciting cause, breaking down the resistive power of a patient predisposed by heredity to the infection of this type of disease." The delusional (chronic) alcoholic cases never exhibited any leucocytosis whatever. (*g*) In most cases of hebephrenia the leucocytosis was 12,000 to 14,000, but every now and then they presented a marked hyperleucocytosis, due to a special increase of the hyaline or large mononuclear cells, which varied between 20 and 30 per cent. (*h*) All cases of paranoia or of delusional insanity occurring in later life were entirely free from hyperleucocytosis. (*k*) The leucocytosis of general paralysis varied according to the case. At the outset and in a classical case there was always hyperleucocytosis and a high polymorphonuclear percentage, and the higher the leucocytosis the more marked was the apparent remission which followed the attack. During the second stage of general paralysis the leucocytosis followed the course of the disease, febrile attacks being indicated by rise of the percentage of leucocytes and of polymorphonuclear cells. Transient eosinophiles were quite common during the second stage of the disease. (*l*) In epileptic insanity hyperleucocytosis was invariably present, not only during periods of seizures, but even in the intervals which were free of attacks. The most marked period of hyperleucocytosis followed a series of fits (*status epilepticus*). No cells exhibiting evidences of phagocytosis were ever found in any case.

[1] Jour. of Mental Sci., July 1904.

(*To be continued.*)

